# Helping patients discuss CINV management: development of a Patient Charter

**DOI:** 10.3332/ecancer.2013.296

**Published:** 2013-03-14

**Authors:** Annie Young, Pascale Dielenseger, Paz Fernandez Ortega, Dolores Fernandez Perez, Philippa Jones, Elaine Lennan, Eileen O’Donovan, Sue Sharp, Alison Whiteford, Lilian Wiles

**Affiliations:** 1 Warwick Medical School, University of Warwick, Coventry, UK; 2 Clinical Research and Early Clinical Trials Unit, Institut Gustave-Roussy, Paris, France; 3 Institut Català d’Oncologia, L’Hospitalet, Barcelona, Spain; 4 Complejo Hospitalario de Ourense, Ourense, Spain; 5 Greater Midlands Cancer Network, Wolverhampton, UK; 6 University Hospital Southampton, Southampton, UK; 7 Irish Cancer Society, Dublin, Ireland; 8 Worcestershire Acute Hospitals NHS Trust, Worcester, UK; 9 Macmillan Cancer Support, G2 8BH, Glasgow, UK; 10 Beating Bowel Cancer, Teddington, UK

## Abstract

In April 2012, an Expert Group of specialist cancer nurses working in a variety of settings (e.g. chemotherapy delivery, chemotherapy service design, research, nurse leadership and patient information/advocacy) participated in telephone/web-based meetings, with the aim of sharing current experience of chemotherapy-induced nausea and vomiting (CINV) management, and reaching a consensus on the development of a Patient Charter, designed to help patients understand CINV management, and setting out key questions they may wish to ask their healthcare professionals.

## Introduction

At the outset of chemotherapy, patients frequently cite the risk of nausea and/or vomiting as one of their biggest fears [1]. Without effective management, CINV not only impairs patients’ quality of life [2], it also increases the risk of dehydration, paraesthesia, malnutrition and gastrointestinal trauma [3], and can lead to unscheduled chemotherapy delays, dose reduction or even cessation, with potential implications for treatment efficacy [4–6].

The risk of CINV varies according to the chemotherapy agents delivered. Without adequate prophylactic treatment, highly emetogenic agents such as cisplatin are likely to cause CINV in more than 90% of recipients [1]. In addition, several agents fall into the moderately emetogenic category, associated with a 31–90% risk of CINV [1]. Furthermore, the likelihood of CINV is increased by patient-associated factors, notably female gender, age under 50 years, a tendency to motion sickness, and experience of nausea or vomiting with previous chemotherapy [6]. Hence, a full assessment of an individual’s risk of CINV requires examination of published evidence on the emetogenic status of the chemotherapy agents used, bearing in mind the dose, delivery method, and the effect of multiple-drug regimens, plus the patient’s history. Based on this information, an appropriate antiemesis strategy can be chosen by a multidisciplinary team of physicians, nurses and pharmacists with experience in cancer care [7]. The use of a risk score to guide the choice of antiemetic therapy is currently being investigated in a randomised controlled trial in women with breast cancer [8].

Evidence based guidelines on the management of CINV have been published (and are regularly updated) by the Multinational Association of Supportive Care in Cancer in association with the European Society of Medical Oncology (MASCC/ESMO), and also by the National Comprehensive Cancer Network (NCCN), and the American Society of Clinical Oncology (ASCO) [9–11]. All of these documents highlight the key role of CINV prophylaxis, administered from chemotherapy cycle 1 before any symptoms of nausea or vomiting appear, for patients deemed to be at high or moderate risk. However, there is a perception among oncology nurses and patient advocates that the management of CINV for some patients, does not meet the standards set out by these guidelines. This perception is supported by the findings of a prospective multicentre community based study published in 2012, which found that 68% of 277 chemotherapy recipients experienced nausea and 23% reported vomiting two to five days after their first treatment cycle [12]. While patients who developed CINV on the day of their treatment were likely to receive additional antiemetic treatment, symptoms that developed after two to five days did not influence CINV management.

This article describes the outcomes of a telephone/web-based nurse consensus meeting convened to share experience and views on current management of CINV, and to agree on the formulation of a Patient Charter, designed to inform chemotherapy recipients about CINV management, and help them to discuss preventative and interventional strategies with their oncology healthcare professionals.

## Methodology

Three virtual meetings were held in April and May 2012, facilitated by a web-based slide presentation from the Chair, Annie Young, and a conference call facility. The presentation included evidence for patients’ perceptions of chemotherapy side effects, published antiemetic guidelines and the limitations of clinical practice guidelines. The proceedings were minuted by a medical writer. All group members participated in the development of the resulting consensus and Patient Charter.

## Unmet need

In practice, healthcare professionals may not witness episodes of CINV. As well as acute nausea and vomiting (occurring within 24 h of the chemotherapy dose, with a peak of intensity after five to six h), many patients experience delayed CINV in the days following their treatment, and symptoms are often at their worst at a time when the patient is at home, rather than in the clinic [6].

**Figure 1:**
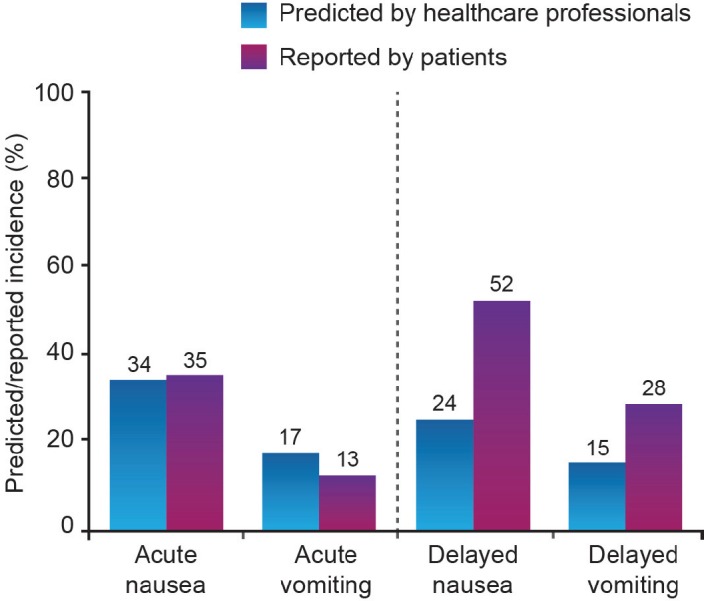
Professional prediction of CINV risk versus patients’ experience following chemotherapy [13]

Possibly as a result of CINV developing outside of the treatment setting, there is a tendency for healthcare professionals to underestimate the risk of CINV (Figure 1) [13–15].

The Expert Group agreed that it is difficult to determine to what extent the poor patient experience, relative to professional expectations, is driven by failure to fully assess the individual’s risk of CINV, failure to provide appropriate and timely CINV prophylaxis based on the findings of the assessment, or patients’ lack of compliance with their prescribed antiemetic regimen.

## Consensus statement: Effective, guideline based management of CINV is not offered to all eligible patients, and the reasons are potentially multifactorial

### Impact of nausea

The Expert Group considered that nausea is often more distressing and debilitating for patients than vomiting; a view supported by the literature, which shows that nausea has come first or second in patients’ ranking of side effects, and ahead of vomiting for several years (Table 1) [16]. In part, the group felt that the preeminence of nausea versus vomiting reflected the greater efficacy of current CINV management in terms of vomiting versus nausea. In addition, the group felt that prolonged nausea without the ‘relief’ of vomiting was *per se *damaging to patients’ quality of life. However, the Expert Group decided not to address nausea and vomiting separately in either the consensus or the Patient Charter, because the vast bulk of the evidence base addresses CINV as a combined entity.

## Consensus statement: Nausea is often more distressing and debilitating than vomiting, and appears to be less effectively managed by current antiemetic regimens

### CINV management

### International guidelines

The Expert Group endorsed the recommendations of CINV management guidelines published by MASCC/ESMO, NCCN and ASCO [9–11], which are evidence based, up-to-date, and focus primarily on CINV prevention rather than symptom management. The group also welcomed the high level of concordance in the advice provided by these three different organisations. However, it was noted that the international guidelines are not always applied at local level. For example, some chemotherapy services (e.g. in the UK) follow locally devised guidance that excludes higher cost drugs (because of spending constraints), and some services do not always assess all patient related CINV risk factors at cycle 1 of chemotherapy (because of time constraints). Also, the MASCC/ESMO, NCCN and ASCO guidelines focus largely on pharmaceutical management of CINV, with scant information or advice on additional approaches that patients may find helpful. There are some reports in the literature of non-pharmacological treatment for CINV, e.g. use of ginger [17, 18]. The Expert Group also cited relaxation techniques and distraction strategies as potential non-pharmacological approaches.

**Figure 2.**
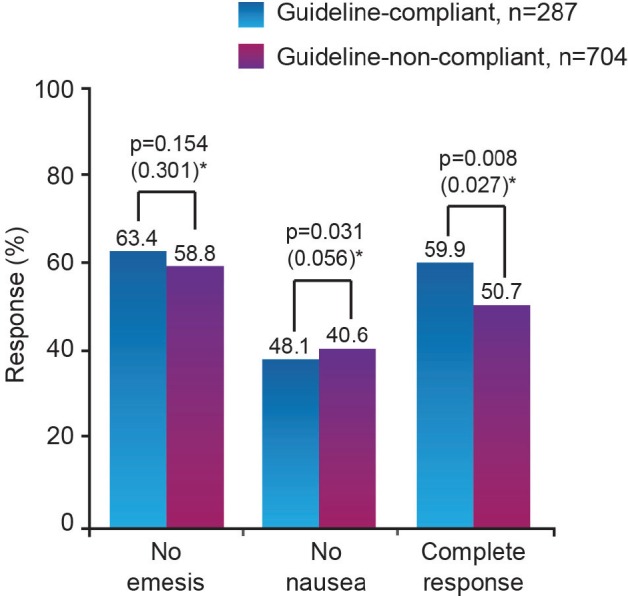
Correlation between guideline compliance and emesis/nausea control in cycle 1 of chemotherapy [19]. (*p values derived by Chi-square test and (in brackets) from multivariate model adjusted for age, sex, pre-chemotherapy anxiety, expectation of nausea, use of primary antiemetic therapy not recommended by guidelines, underdosing of primary antiemetic therapy, and use of secondary antiemetic agents)

**Figure 3.**
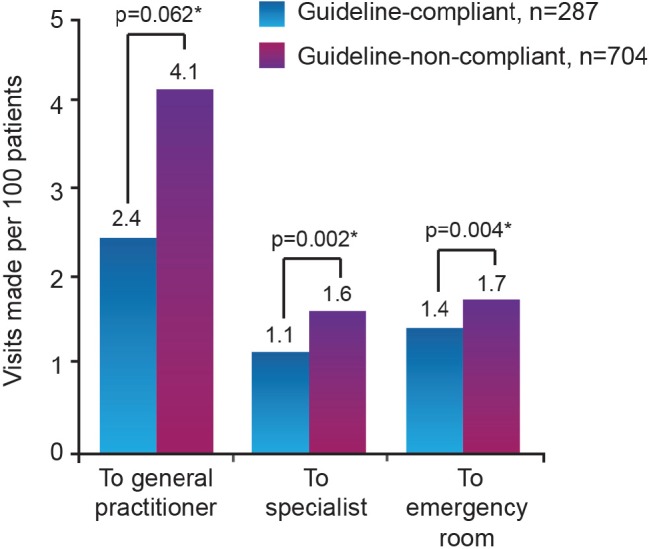
Correlation between guideline compliance and healthcare visits for CINV in the five days after cycle 1 of chemotherapy  [19]. (*p values derived from multivariate model adjusted for age, sex, pre-chemotherapy anxiety, expectation of nausea, use of primary antiemetic therapy not recommended by guidelines, underdosing of primary antiemetic therapy and use of secondary antiemetic agents)

The efficacy of antiemetic practice that closely follows the MASCC CINV recommendations (referred to as ‘guideline-compliant’) has been demonstrated by the Pan European Emesis Registry study, conducted in Austria, Belgium, France, Italy, Spain, Sweden, The Netherlands and the UK. This research showed that 60% of patients undergoing highly or moderately emetogenic chemotherapy in guideline-compliant centres remained emesis-free (including no use of rescue medication) for five days following cycle 1 of highly or moderately emetogenic chemotherapy, compared with 51% of those in non-compliant centres (p = 0.008) (Figure 2) [19]. Furthermore, the patients in the guidelinecompliant group made fewer CINV related visits to specialist or emergency hospital care during the five days following the initiation of their first chemotherapy cycle, compared with those in the non-compliant group (Figure 3).

## Consensus statement: National and international guidelines are a useful starting point for CINV policy, and offer a strong evidence base for the pharmaceutical aspects of care, but lack detail on complementary and psychological strategies, and are not universally applied

### Patient assessment and tailored care

The Expert Group emphasised the importance of comprehensive patient assessment with proactive questioning about potential CINV risk factors, to be conducted before chemotherapy cycle 1 and then again before each subsequent cycle. Information from this assessment in combination with the published emetogenicity of the specific chemotherapy regimen, should be used to tailor the individual patient’s CINV management. Reliance solely on the emetogenicity of the chemotherapy regimen without taking account of individual patient factors such as age, sex, previous experience of nausea/vomiting, and history of CINV can result in an underestimation of the level of CINV risk, and hence inadequate provision of prophylaxis. The group suggested that nurses may often be better placed than doctors to assess the patients’ risk factors for CINV, because they frequently conduct a holistic assessment prior to chemotherapy and may have more frequent contact with individual patients.

## Consensus statement: Assessment of CINV risks should include patient factors as well as the recorded emetogenicity of the chemotherapy regimen

### Patient education/information

It is important to ensure the patient understands the strategy of CINV management, including the nature of prophylaxis versus symptomatic treatment and the need to take prescribed antiemetic medication as instructed, even if there is no nausea or vomiting.

## Consensus statement: It is essential to explain to patients that prophylactic medications must be taken as prescribed, regardless of how well they feel

Given the variation between centres in the implementation of guideline based management, the Expert Group recommended using the Patient Charter to empower patients to enquire about the basis of local CINV practice.

## Consensus statement: The Patient Charter may help to empower patients to question healthcare professionals about local use of CINV guidelines

The Expert Group also advised healthcare professionals to encourage open discussion about complementary remedies for nausea and vomiting. Proactive questioning may be helpful; some patients hesitate to mention such interventions, perhaps because they do not think it is relevant to their consultation, or out of fear that the health professional will disapprove of alternative treatments. It is important to ensure that the complementary remedy will do no harm, e.g. through interaction with the chemotherapy regimen, the antiemetic drugs, or any other concomitant medication. It is also important to stress that it should be regarded as an additional treatment rather than an alternative to the antiemetics, i.e. patients must not stop taking their prescribed medication. In practice, the Expert Group members were happy to support and advise patients who wished to try complementary approaches to the management of nausea and vomiting, and to provide information on some of the methods available. Some chemotherapy clinics may be able to offer referral to local complementary practitioners.

## Consensus statement: There needs to be open dialogue about use of complementary remedies

### Monitoring and follow up

The Expert Group identified two essential aspects to CINV management between scheduled clinic visits, i.e. accurate symptom monitoring and timely intervention where required.

### Symptom monitoring

If CINV develops between chemotherapy cycles, the symptoms have typically abated by the time the next cycle is due. Based on their clinical experience, the group members said that patients frequently underplay the severity of their symptoms, partly because they have no CINV on the day of their consultation, but sometimes also out of a wish to avoid ‘troubling’ the healthcare professional. Furthermore, patients may fear their forthcoming chemotherapy might be delayed, cancelled or reduced in dose if they disclose side effects. In a survey-based study published in 2012, 37% of patients said they wanted to appear “strong by not complaining” [14]. Without accurate information on the patient’s experience on CINV, it is difficult to offer a tailored antiemetic programme. The Expert Group recommended real-time recording of CINV, e.g. using a patient-held side effect diary (hard copy or in the form of a mobile phone app). Proactive management at this stage could include phone calls from the chemotherapy clinic on days when the patient is predicted to be particularly susceptible to nausea and/or vomiting.

## Consensus statement: Real-time monitoring, e.g. using diaries or proactive follow-up phone calls, is essential to obtain an accurate record of symptoms between clinic appointments

### Management of symptoms

Patients should be encouraged to seek help for all but the very mildest nausea or vomiting, and to do so as soon as symptoms develop. Patients should not wait to see if their symptoms resolve spontaneously, or wait until clinic opening times if they develop nausea or vomiting. The Expert Group advised providing every patient with clear contact information, showing who to contact and what phone number to use during and outside clinic hours, and emphasising the importance of making contact straight away.

## Consensus statement: Patients need clear contact information for reporting the development of nausea or vomiting, and instructions to seek help straight away

### Professional education needs

The Expert Group called for all professionals involved in the care of patients receiving chemotherapy to receive education on the management of CINV and the importance of rapid, appropriate intervention. Such education should be extended to primary as well as secondary care, and to staff in emergency departments.

### Patient Charter

**Figure 4.**
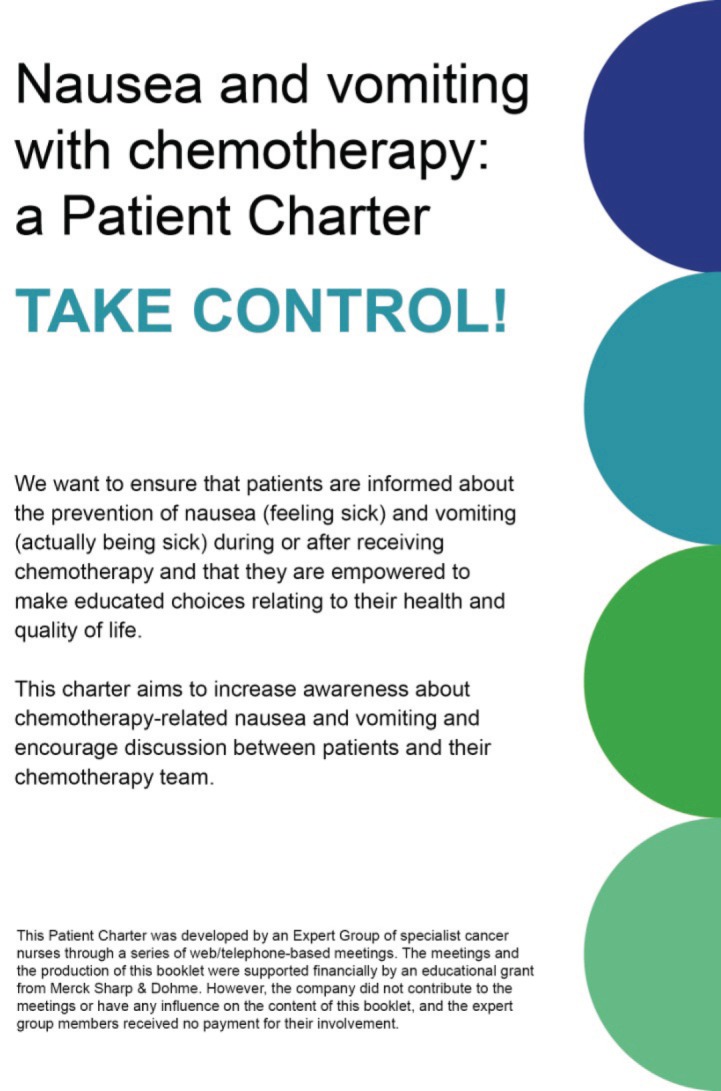
Patient Charter on CINV

The Expert Group has developed a Patient Charter on CINV (Figure 4), which provides information on control of nausea and vomiting and suggests questions that the patient may wish to ask their healthcare professional. It is proposed that the Charter will be disseminated to patients via oncology clinics, hospices, general practice surgeries, cancer charities, professional oncology groups, patient associations, patient-advocacy groups and other appropriate organisations.

## Consensus statement: The Patient Charter on CINV management, based on the discussions of the Expert Group, and including a prompt sheet of key questions, will help to educate patients and empower them to question decisions surrounding their own care

## Conclusion

Despite the advances in CINV management, and the availability of regularly updated international guidelines, not all patients receive the interventions they need to prevent this common side effect. The consensus statements, i.e. the key observations and recommendations of the Expert Group, are as follows:

- Effective, guideline based management of CINV is not offered to all eligible patients, and the reasons are potentially multifactorial
- Nausea is often more distressing and debilitating than vomiting, and appears to be less effectively managed by current antiemetic regimens
- National and international guidelines are a useful starting point for CINV policy, and offer a strong evidence base for the pharmaceutical aspects of care, but lack detail on complementary and psychological strategies, and are not universally applied
- Assessment of CINV risks should include patient factors as well as the recorded emetogenicity of the chemotherapy regimen
- It is essential to explain to patients that prophylactic medications must be taken as prescribed, regardless of how well they feel
- The Patient Charter may help to empower patients to question healthcare professionals about local use of CINV guidelines
- There needs to be open dialogue about use of complementary remedies
- Real-time monitoring, e.g. using diaries or proactive follow up phone calls, is essential to obtain an accurate record of symptoms between clinic appointments
- Patients need clear contact information for reporting the development of nausea or vomiting, and instructions to seek help straight away
- The Patient Charter on CINV management, based on the discussions of the Expert Group, and including a promptsheet of key questions, will help to educate patients and empower them to question decisions surrounding their own care

The Expert Group hopes that the Patient Charter will help encourage effective dialogue between chemotherapy recipients and their healthcare professionals on CINV and its management.

## Figures and Tables

**Table 1.** How patients rank the severity of chemotherapy side effects [16]

Rank	1996	1997	1999	2004
1	Nausea	Nausea	Nausea	Fatigue
2	Constantly tired	Hair loss	Hair loss	Nausea
3	Hair loss	Vomiting	Constantly tired	Sleep disturbances
4	Effect on family	Constantly tired	Vomiting	Weight loss
5	Vomiting	Injections	Taste changes	Hair loss
